# Personalized Interactive Music Systems for Physical Activity and Exercise: Exploratory Systematic Review and Meta-Analysis

**DOI:** 10.2196/70372

**Published:** 2025-09-08

**Authors:** Andrew Danso, Tiia Kekäläinen, Friederike Koehler, Keegan Knittle, Patti Nijhuis, Iballa Burunat, Pedro Neto, Anastasios Mavrolampados, William M Randall, Niels Chr Hansen, Alessandro Ansani, Timo Rantalainen, Vinoo Alluri, Martin Hartmann, Rebecca S Schaefer, Johanna K Ihalainen, Rebekah Rousi, Kat R Agres, Jennifer MacRitchie, Petri Toiviainen, Suvi Saarikallio, Sebastien Chastin, Geoff Luck

**Affiliations:** 1 Department of Music, Arts and Culture Studies, Centre of Excellence in Music, Mind, Body and Brain, University of Jyväskylä, Seminaarinkatu 15, Jyväskylän yliopisto, Jyväskylä, 40014, Finland, 358 6643034; 2 Laurea University of Applied Sciences, Vantaa, Finland; 3 Gerontology Research Center and Faculty of Sport and Health Sciences, University of Jyväskylä, Jyväskylä, Finland; 4 Faculty of Sport and Health Sciences, University of Jyväskylä, Jyväskylä, Finland; 5 Royal Academy of Music Aarhus, Aarhus, Denmark; 6 Cognitive Musicology and Performance Science Lab, Department of Communication and Psychology, Aalborg University, Aalborg, Denmark; 7 Cognitive Science Lab, International Institute of Information Technology, Hyderabad, India; 8 Health, Medical, and Neuropsychology Unit, Institute of Psychology, Faculty of Social and Behavioural Sciences, Leiden University, Leiden, The Netherlands; 9 Leiden Institute for Brain and Cognition, Leiden University, Leiden, The Netherlands; 10 Academy of Creative and Performing Arts, Faculty of Humanities, Leiden University, Leiden, The Netherlands; 11 Finnish Institute of High Performance Sport KIHU, Jyväskylä, Finland; 12 School of Marketing and Communication, Communication Studies, University of Vaasa, Vaasa, Finland; 13 Centre for Music and Health, Yong Siew Toh Conservatory of Music, National University of Singapore, Singapore, Singapore; 14 Department of Music & Healthy Lifespan Institute, University of Sheffield, Sheffield, United Kingdom; 15 School of Health and Life Sciences, Glasgow Caledonian University, Glasgow, United Kingdom; 16 Department of Movement and Sports Sciences, Ghent University, Ghent, Belgium

**Keywords:** music intervention, health promotion, exercise, affect, systematic review, meta-analysis, mobile phone

## Abstract

**Background:**

Personalized Interactive Music Systems (PIMSs) are emerging as promising devices for enhancing physical activity and exercise outcomes. By leveraging real-time data and adaptive technologies, PIMSs align musical features, such as tempo and genre, with users’ physical activity patterns, including frequency and intensity, enhancing their overall experience.

**Objective:**

This exploratory systematic review and meta-analysis evaluates the effectiveness of PIMSs across physical, psychophysical, and affective domains.

**Methods:**

Searches across 9 databases identified 18 eligible studies, of which 6 (comprising 17 intervention arms) contained sufficient data for meta-analysis. Random-effects meta-analyses and meta-regression were performed to assess outcomes for physical activity levels, physical exertion, ratings of perceived exertion, and affective valence.

**Results:**

Results showed significant improvements in physical activity levels (*g*=0.49, CI 0.07 to 0.91, *P*=.02, *k*=4) and affective valence (*g*=1.65, CI 0.35 to 2.96, *P*=.01, *k*=4), with faster music tempo identified as a significant moderator (*P*=.03). No significant effects were observed for ratings of perceived exertion (*g*=0.72, CI −0.13 to 1.58, *P*=.10, *k*=3) or physical exertion (*g*=0.78, CI −0.55 to 2.11, *P*=.25, *k*=5).

**Conclusions:**

Substantial heterogeneity and limited study quality indicate the need for more robust, randomized controlled trials to establish the efficacy of PIMSs in diverse populations.

## Introduction

### Background

Regular physical activity and exercise are fundamental to maintaining and enhancing overall health and well-being. Despite their recognized role in preventing and managing noncommunicable diseases such as cardiovascular diseases, cancer, and diabetes, engagement in regular physical activity and exercise remains below the suboptimal level [1]. This deficiency undermines the potential for mental health benefits of physical exercise and its contributions to quality of life [2]. The World Health Organization defines physical activity broadly, encompassing all forms of bodily movement generated by skeletal muscles that require energy expenditure, including activities such as walking, sports, and dance [1]. In contrast, exercise has been defined as “physical activity that is planned, structured, repetitive, and purposive, aiming to improve or maintain one or more components of physical fitness” [3]. However, the broad spectrum of activities categorized as physical activity and exercise often presents challenges in promoting consistent engagement and uptake, including individual-level barriers such as motivation and time constraints [4]. Efforts to increase engagement in physical activity and exercise have faced significant challenges, frequently yielding inconsistent outcomes, as exemplified by interventions such as pedometer-based programs, which demonstrate variable effectiveness depending on factors including participant motivation and engagement [4,5].

### Role of Music in Enhancing Physical Activity and Exercise

Music’s rhythmic properties have been shown to influence perceptions, ergonomics, and physiological markers associated with physical activity and exercise [6-10]. Available evidence suggests that auditory-motor coupling facilitates predictive synchronization in physical activity and exercise settings, which can reduce perceived exertion and enhance endurance [11,12]. Additionally, when music aligns with individual preferences, such as through self-selection, it may further increase motivation, improve affective states, induce distraction, and lower perceived effort during physical activities and exercise [7,11].

The integration of music into exercise contexts can be further understood through theoretical frameworks such as the Affective-Reflective Theory (ART) and Dual-Mode Theory. ART emphasizes the importance of momentary affective responses—such as pleasure or displeasure—in shaping future exercise behaviors [13,14]. These responses, encapsulated in the construct of “affective valence,” reflect the intrinsic pleasantness or unpleasantness of emotional states that fluctuate in response to internal and external stimuli. Conversely, Dual-Mode Theory posits that music’s impact on affective responses is most pronounced at moderate exercise intensities, within a zone of response variability. This zone refers to the range of exercise intensity where affective responses—such as feelings of pleasure or displeasure—are particularly sensitive to individual differences (eg, fitness level and psychological state) and contextual factors (eg, music, environment, and social setting). In this range, attentional focus and physiological cues mediate affective experiences [15]. While both theories acknowledge the importance of affective responses in exercise, Dual-Mode Theory provides a more nuanced perspective by emphasizing intensity-dependent variability and its interaction with individual and contextual factors.

Extending the principles of ART and Dual-Mode Theory [16], the framework highlights how music’s intrinsic properties—such as tempo, rhythm, and harmony—interact with personal and situational moderators, including exercise intensity and individual preferences, to influence affective and behavioral outcomes in exercise. Music operates through 3 primary mechanisms: regulating affective states, dissociating attention from exertional discomfort, and facilitating temporal prediction and rhythmic synchronization. These mechanisms are most effective within the zone of response variability, where affective valence dynamically influences exercise engagement [15,17]. Empirical studies consistently demonstrate that personalized music enhances energy efficiency, reduces perceived exertion, and improves adherence by fostering positive affect [17]. Such evidence positions personalized music systems as a key tool for optimizing both the immediate and long-term benefits of exercise.

### Personalized Interactive Music Systems in Physical Activity and Exercise

Recently, advances in personalized music technologies have led to the development of Personalized Interactive Music Systems (PIMSs), which leverage software, sensors, and computer algorithms to deliver a dynamic, tailored music experience during physical activity and exercise [18,19]. These systems integrate with smartphones and wearable devices to monitor user movements and adjust musical features, such as tempo, style, and timbre, in real time to align with exercise routines, enhancing engagement and adherence to activity [20,21].

PIMSs have been designed for diverse contexts, targeting both intrinsic factors (eg, motivation and attentional focus) and extrinsic factors (eg, training guidance). For example, a PIMS, the moBeat system, used real-time interactive music and biophysical feedback to enhance cycling performance by increasing intrinsic motivation and maintaining pace and intensity [12]. Similarly, PIMS interventions for older adults have demonstrated benefits for physical endurance and engagement relative to conventional exercise conditions [22]. As mobile interventions incorporating personalization have been shown to be more effective at enhancing physical activity than nonpersonalized approaches [23], PIMSs hold promise for improving physical activity adherence, reducing the ratings of perceived exertion (RPE), and fostering positive affective states during exercise by dynamically tailoring music to individual physiological, affective, and contextual needs [7,12,22].

Due to the relatively recent advancements of PIMSs, there is yet limited empirical evidence on their effectiveness across physical activity and exercise-related domains. Such information is essential for informing implementation, replication, and comparative evaluation of interventions aimed at promoting adherence to physical activity and exercise [24]. While systematic reviews and meta-analyses have explored the general effects of music on physical activity and exercise-related outcomes [6,7,25], these reviews predominantly focused on traditional music listening interventions and did not systematically evaluate the impact of personalized and interactive music systems. By specifically examining PIMSs, this review and meta-analysis contribute to understanding how tailored, interactive music interventions influence physical, psychophysical, and affective dimensions of physical activity and exercise engagement, thereby addressing a critical gap in the existing literature.

Therefore, this study combines a systematic review and exploratory meta-analysis to evaluate the effectiveness of PIMSs on physical activity and exercise-related outcomes. Specifically, this study synthesizes findings on physical activity levels, psychophysical measures (eg, RPE and physical exertion), and affective outcomes (eg, affective valence and mood states).

Our main research question is: How effective are PIMSs across physical, psychophysical, and affective outcomes during physical activity and exercise? This analysis intends to provide early insights into the specificity of PIMSs’ effects and identify gaps in the literature that warrant further investigation.

## Methods

### Study Design

This systematic review and meta-analysis were designed based on the PRISMA (Preferred Reporting Items for Systematic Reviews and Meta-Analyses) protocol [26]. The full search strategy can be found in the review registration document (CRD42023465941).

### Eligibility Criteria

We included (1) studies investigating the effect of PIMSs on physical activity or exercise, including their effects on motivation, exercise intensity, adherence, or related outcomes, (2) studies including participants from diverse populations (eg, sufficiently active and not sufficiently active individuals), and (3) papers in the English language, published from January 2010 to May 2024 in peer-reviewed journals or as published proceedings (conference papers were considered due to the limited number of peer-reviewed studies).

We excluded (1) studies from nonpeer-reviewed sources, books, dissertations, and theses; (2) papers written in languages other than English; and (3) studies that were not directly related to the effect of PIMSs on physical exercise or physical activity.

### Information Sources

We searched the following databases: (1) Web of Science, (2) SPORTDiscus, (3) Medline, (4) Embase, (5) ACM Digital Library databases, (6) Springer, (7) Google Scholar, (8) IEEE Xplore, and (9) Scopus. The database search was supplemented by a backward snowball search, whereby the reference list of all papers was scanned for potential sources. The snowball search continued until no new sources could be identified. The initial interrater agreement for the identification of relevant sources was *k*=0.83, indicating a strong level of agreement among the 2 individuals performing 2 independent snowball searches (AD and TK). Full search strings for all databases used in this review are provided in Section S3 in Multimedia Appendix 1.

### Search Strategy

A literature search was performed using terminology related to the effects of PIMSs on physical activity and exercise, (“Personali*ed Interactive Music System*” OR “Music Recommendation Algorithm” OR “Music Recommendation System*” OR “Streaming” OR “MP3” OR “Digital Music”) AND (“Physical Activity” OR “Exercise” OR “Recovery” OR “Recuperation” OR “Sedentary Behav*” OR “Physical Inactivity”).

### Selection Process and Data Collection Process

The citations of all retrieved papers were imported into Zotero (Digital Scholar), where duplicates were systematically identified and removed. Subsequently, 2 authors (AD and TK) independently screened the titles and abstracts of the studies using ASReview (Utrecht University) [27] and Rayyan [28]. Papers that could not be definitively excluded based on the title or abstract underwent full-text retrieval for further evaluation. The full-text papers were then independently assessed for inclusion by the same 2 authors (AD and TK). Disagreements at any stage were resolved through discussion, with a third author consulted to achieve consensus when necessary.

### Data Extraction

The studies’ information was extracted to a spreadsheet, including study characteristics, such as the type of PIMS, the study design, PIMS measurement, and the target behavior of the PIMS (Target Physical Activity or Exercise; Table 1).

**Table 1.** Characteristics of included studies.

Reference	Country	Age (years)	Sample size (N)	Population	Type of PIMS^a^	Study design	PIMS measurement	Target behavior or target physical activity or exercise	Physical activity results	*G* (95% CI) of PIMS on outcomes of interest
[29]	Canada	47.3‐79.2^b^	34	Patients with cardiovascular disease	Personalized music audio-playlists	Randomized experimental design	Triaxial accelerometer	Adherence	Improved PA^c^ volumes (*P*<.001)	*g*=0.51 (−0.47 to 1.49) for physical activity level (RAS^d^) *g*=−0.06 (−1.04, 0.92) for physical activity level (no RAS)
[30]	Spain	N/A^e^	N/A	N/A	Personalized music recommendation system	Proof of concept	Sensors^f^	Motivation or performance enhancement	N/A	N/A
[31]^g^	Germany	N/A	1	Older adult participant	Music feedback for rehabilitation	Proof of concept	Accelerometer	Rehabilitation	N/A	N/A
[32]^g^	Taiwan	21.56 (SD 1.04)^h^	10 female and 26 male	Participants from the National Yang Ming Chiao Tung University	Exercise system for middle-distance running	Experimental	Smartphone’s built-in triaxial accelerometer	Adapting music selection to the user’s pace during walking	N/A	*g*=−0.73 (−1.40 to −0.06) for physical exertion *g*=1.63 (0.88 to 2.39) for RPE^i^ *g*=2.17 (1.34 to 2.99) for affective valence
[33]	Taiwan	N/A	N/A	N/A	Music assisted run trainer	Proof of concept	Triaxial accelerometer	Physiological, perceptual, and affective responses	N/A	N/A
[34]^g^	Singapore	N/A	60	Students	A music recommendation system	Within-subjects crossover design	Music recommendation ratings	Motivation	N/A	N/A
[35]	N/A	N/A	45	Nonathletes, nonbody builders, nonmusicians	JYMMiN sensor attached to fitness devices to provide musical feedback	Experimental design	Movement sensor^j^	Workout	N/A	*g*=0.96 (0.35 to 1.56) for affective valence
[36]^g^	N/A	N/A	27	N/A	Runner’s Jukebox: music tempo matching the user’s pace during exercise	User testing design	Smartphone app to recognize user pace or adjust music tempo	Walking or running pace monitor^k^	N/A	*g*=2.76 (1.63 to 3.89) for physical exertion (fixed BPM^l^) *g*=2.29 (1.24 to 3.34) for physical exertion (pace-matched) *g*=−0.74 (−1.61 to 0.13) for physical exertion (random)
[37]^g^	Denmark	N/A	N/A	N/A	Music clips with dynamic BPM ranging from 110‐170	Proof of concept	Sensors^m^	Cycling	N/A	N/A
[38]	Switzerland	18‐45	7 females and 8 males	Cyclists	SoundBike: musical sonification to improve spontaneous synchronization of cyclists	Experimental	Sensors	Cycling	Enhanced cyclist synchronization to external music	N/A
[39]	Finland	N/A	2	Older adult participants	Processing accelerometry data to create musical sonifications of physical activity	Proof of concept	Sonification of PA data^n^	Awareness of PA	N/A	N/A
[40]^g^	Belgium	N/A	33	Participants from public event	DSaT^o^ algorithm for music selection and real-time adaptation	Pilot study	Triaxial accelerometer	Synchronization to the beat of music	The majority (19/33, 58%) synchronized their steps with music	N/A
[41]	Belgium, Czech Republic	21.9 (SD 12.9)^h^ 20.2 (SD 0.8)^h^ 21.2 (SD 1.7)^h^ 23 (SD 3)^h^	82 male and 68 female 56 male and 44 female 12 female 6 female and 4 male	N/A	Synchronize music with the participant’s movements	Case study	Recordings of footfalls and music alignment strategies^p^	Synchronization to the beat of music	Improved entrainment	N/A
[42]^g^	N/A	N/A	N/A	N/A	Context-aware recommender system	Mixed methods design ^q^	Automatic learning algorithm	Motivate users to complete PA	N/A	N/A
[22]	Germany	70.6 (SD 3.9)^h^	11 females and 5 males	Nonphysically active	JYMMiN: sensor attached to fitness devices to provide musical feedback	Within-subjects design	Movement sensor^j^	Strength-endurance exercises	N/A	*g*=0.73 (0.00 to 1.46) for physical activity level *g*=0.20 (−0.27 to 0.67) for RPE *g*=0.09 (−0.47 to 0.64) for affective valence
[43]^g^	Netherlands	18‐25	24	Office workers	Smart cushion providing musical feedback	Within-subjects design	Movement sensor pad	Posture changes	No effect on breaking sedentary behavior	N/A
[44]	Norway	N/A	3-6^r^	Seniors with early-stage Alzheimer disease	Interactive music system	Qualitative research design	Sensor pad	Stimulate or motivate PA	N/A	N/A
[12]	Netherlands	23‐51	26	Philips employees	moBeat: interactive music system	Within-subject experiment	Cadence sensor, heart rate	Motivation	N/A	*g*=0.54 (−0.22 to 1.30) for physical activity level *g*=0.54 (−0.22 to 1.30) for physical exertion *g*=0.43 (−0.32 to 1.19) for RPE *g*=3.79 (2.52 to 5.06) for affective valence

a PIMS: Personalized Interactive Music System.

b Lowest lower bound: 47.3 years (from the second subgroup). Highest upper bound: 79.2 years (from the first subgroup). The estimated entire age range for all 3 groups combined would be from approximately 47.3 to 79.2 years.

c PA: physical activity.

d RAS: rhythmic auditory stimulation.

e N/A: not applicable.

f Galvanic skin response, oxygen saturation sensor, and pulse sensor.

g Conference papers.

h Mean (SD).

i RPE: ratings of perceived exertion.

j JYMMiN: the movement of the sensor-equipped fitness device is mapped to musical parameters, creating an acoustic feedback signal.

k SWPM: swings per minute.

l BPM: beats per minute.

m Monitor cycling pace and heart rate, influencing audio feedback (soundscape sounds) in real-time.

n Accelerometry data.

o DSaT: Dynamic Song and Tempo.

p The methodology involved recording footfalls and various music alignment strategies to synchronize music with participants’ walking or running movements.

q Includes elements of a proof of concept design and an experimental design.

r Exact numbers are not specified, but a mention of a group size of 3 to 6 participants.

### Preregistration Deviations

Where available, quantitative data suitable for meta-analysis were extracted. This was done for the preregistered outcome of physical activity level, as well as for affective valence, RPE, and physical exertion, which were not preregistered as outcomes. The decision to extract data on these additional outcomes was taken because of the close relationships between these variables and physical activity and exercise participation, their prevalence as outcomes in the included studies, and the limited number of studies reporting data on physical activity and exercise behavior. In cases where effect sizes could not be readily calculated based on the published papers, their authors (n=2) were contacted at least twice for additional data, resulting in the provision of calculations for 5 additional effect sizes.

### Operationalization of Terms

This review operationalizes 4 key terms central to physical activity and exercise research. Physical activity level is defined by the quantified volume (eg, daily activity counts and weekly minutes), intensity (eg, metabolic equivalent of task [MET] and percent oxygen uptake reserve), and compliance (eg, adherence to heart rate zones or regimens) [45,46] of physical activity. Affective valence refers to the pleasure-displeasure dimension of emotional responses during or after physical activity, assessed using self-report scales such as the Feeling Scale (FS) [47] and the “good versus bad mood” subscale of the Multidimensional Mood Questionnaire (MDMQ) [48]. These measures capture subjective ratings of positivity or negativity without incorporating arousal [14,35,49]. Physical exertion encompasses physiological (eg, heart rate), biomechanical (eg, stride length), and perceptual demands, providing a comprehensive assessment of effort [50]. These constructs serve as the primary outcomes of interest in this review. The constructs are summarized in Table 2 and described further in Section S1 in Multimedia Appendix 1.

**Table 2.** Operationalization of terms.

Term	Definition	Operational metrics	References
Physical activity level	Encompasses the volume, intensity, and compliance with physical activity recommendations or exercise regimens.	Volume: total activity counts per day via accelerometer, mean weekly minutes. Intensity: absolute intensity using metabolic equivalent of tasks, relative intensity as oxygen consumption reserve percentage. Compliance: adherence to recommendations or regimens via changes in volume, device usage, or adherence to heart rate zones.	[45,46]
Physical exertion	Effort exerted to perform physical activity, involving physiological, biomechanical, and perceptual demands.	Physiological: heart rate as an indicator of cardiovascular response. Biomechanical: stride length and pace for activities such as running and walking. Perceptual: integration of physiological and biomechanical cues to assess overall effort.	[50]
RPE^a^	Subjective numerical value reflecting perceived effort during physical activity, integrating sensory cues, and physiological sensations.	Scale: Borg RPE scale for aerobic activities (cycling and running). Category-Ratio Scale: Borg Category-Ratio 10 Scale to measure perceived exertion or other sensations. Responses: local sensations (muscles, skin, and joints) and central factors (cardiopulmonary system).	[7,51,52]
Affective valence	The subjective feeling of pleasure or displeasure experienced during or after physical activity. It is independent of perceived exertion and reflects emotional responses to exercise, influenced by individual, contextual, and social factors.	Affective valence is measured using self-report scales, such as: Scale: Feeling Scale: bipolar scale from +5 (very good) to −5 (very bad). Positive and negative affect schedule: assesses positive and negative emotions. Multidimensional Mood Questionnaire (MDMQ): evaluates mood during exercise using subscales for “good versus bad mood,” “calmness versus agitation,” and “alertness versus tiredness.” Only the “good versus bad mood” subscale aligns with the pleasure-displeasure dimension of affect. Context: Measurement occurs before, during or immediately after exercise.	[14,47-49]

a RPE: ratings of perceived exertion.

### Study Risk of Bias Assessment

The quality of the studies was assessed by 2 authors (AD and TK) using the JBI’s (Joanna Briggs Institute) critical appraisal checklist, including tools for quasi-experimental appraisal, qualitative research appraisal, and the revised checklist for randomized controlled trials [53].

### Data Synthesis and Analysis Methods

We conducted a narrative synthesis, categorizing studies into two groups based on design: (1) experimental studies, including randomized, quasi-experimental, pilot, and within-subject designs, and (2) proof-of-concept and user-testing studies. This classification enabled the identification of trends within and across these categories.

For experimental studies, we examined outcomes related to physical activity levels, physical exertion, RPE, and affective valence. Proof-of-concept and user-testing studies were analyzed for their focus on PIMS design features and effectiveness, including synchronization, user engagement, and personalization.

Our synthesis followed the methodological framework of [54], facilitating systematic comparisons across study groups. Trends and variations in PIMSs’ outcomes were interpreted through subgroup analyses, accounting for methodological rigor and study design. We also considered sample characteristics, including demographic variability (eg, age, fitness level, and population type) and sample size heterogeneity (ranging from n=10 to n=150). Limitations arising from study heterogeneity were explicitly addressed to provide transparency regarding factors affecting generalizability.

Hedges *g* effect sizes and SEs were calculated using the tool by Wilson [55]. Meta-analytic models were conducted in *R* (version 4.5.1) using the *metafor* package [56], applying a random-effects model with the DerSimonian-Laird estimator for physical activity level, physical exertion, RPE, and affective valence. These outcomes were selected based on the preregistration criterion: “meta-analyses will be performed when at least three studies provide data sufficient for effect size calculation.” For inclusion in the meta-analysis, physical activity outcomes analyzed included behaviors such as walking, running, weight training, cycling, housework, and gardening, while studies focusing on nonphysical activity outcomes (eg, subjective feasibility of PIMSs) were excluded. Six studies (comprising 17 intervention arms) met this criterion, while outcomes with insufficient data were excluded.

Heterogeneity was assessed using the *I*² statistic (relative proportion of variability attributable to heterogeneity), *τ*² statistic (absolute variance), and Cochran *Q* statistic (a formal test of homogeneity). To address the dispersion of effects across studies, the prediction interval was calculated, as it provides insights into the range of effects expected in future comparable studies, beyond the mean effect size [57]. A sensitivity analysis was conducted to evaluate publication bias by examining the relationship between SEs and effect size estimates. Following the studies by Sterne and Egger [58] and Sterne and Harbord [59], funnel plots were produced to assess asymmetry, while forest plots were used to summarize the data. Data and syntax files for these analyses are available in Multimedia Appendix 2.

An exploratory meta-regression analysis was conducted to investigate potential moderators contributing to variability in the effectiveness of PIMSs on physical activity level, affect, RPE, and physical exertion. Candidate moderators were selected based on their theoretical relevance to physical activity and exercise research: study size, participant age, exercise intensity, and music tempo. Music tempo was categorized into tempo ranges to standardize data across studies with differing methodologies, reflecting its established influence on motivational and psychophysical responses [60]. Exercise intensity was classified using MET guidelines to enhance comparability [61]. Participant age and study size were included to address population-level and methodological variability, respectively. Due to the small number of studies included in the meta-analyses, the meta-regression encompassed all outcomes of interest, with a focus on generating hypotheses for future research.

Specifically, within the meta-regression analysis, music tempo was categorized into 3 distinct groups based on beats per minute (BPM): slow (60‐90 BPM), coded as 1; medium (91‐130 BPM), coded as 2; and fast (131+ BPM), coded as 3. When studies reported variable tempos, the average BPM or the dominant tempo range was used for classification. Exercise intensity was categorized using a 3-level scale aligned with MET guidelines [61]: low (<3 METs), coded as 1; moderate (3‐6.9 METs), coded as 2; and high (≥7 METs), coded as 3. For studies that did not explicitly report METs, intensity was inferred from descriptions of the exercise type or target heart rate zones. Participant age was handled as follows: for studies reporting mean age directly, the provided value was used. In studies reporting age ranges, the midpoint of the range was used as an estimate. If group-specific mean ages were available, a weighted average was calculated based on group sizes to derive an overall mean age for the study.

## Results

### Study Selection

All records were excluded by ASReview [27,28].

**Figure 1.**
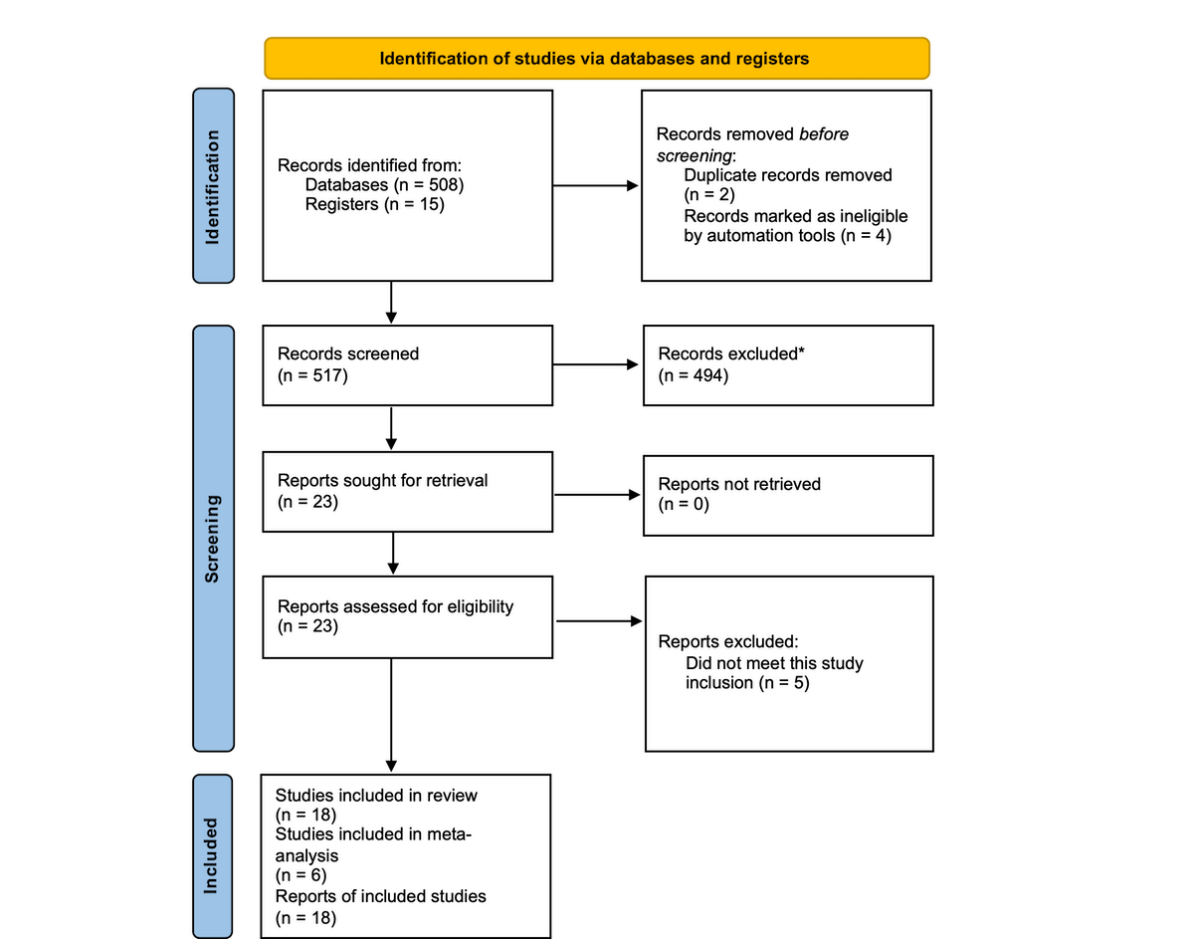
PRISMA information flow describing the screening process. PRISMA: Preferred Reporting Items for Systematic Reviews and Meta-Analyses.

A total of 523 papers were identified through the initial strategic search using the specified keywords. During the screening process, 3 papers were excluded as duplicates, while 4 additional papers were identified as ineligible based on the inclusion criteria. After screening titles and abstracts, 494 papers were excluded for not meeting the inclusion criteria. Subsequently, 23 full-text papers were assessed for eligibility. Of these, 5 papers were excluded [21,62-65] because they did not evaluate the desired effect or outcome. In total, 18 papers were eligible to be included in this review study (Figure 1).

### Study Characteristics

The study characteristics (Table 1) encompass a diverse range of studies conducted across various countries, including Canada, Spain, Germany, Taiwan, Singapore, Denmark, Finland, Belgium, Switzerland, the Czech Republic, the Netherlands, Norway, and locations not specified. These studies, conducted between 2010 and 2024, provide a broad age range among participants, with some studies focusing on specific groups such as older adults, patients with cardiovascular disease, students, nonathletes, and office workers. The PIMSs used in these studies vary in their design and objectives, ranging from personalized music audio playlists [30,34,42] to interactive music systems linked to fitness devices [22,35]. These systems are used in different settings and for various purposes, ranging from synchronizing movement during physical activity and exercise to enhancing the experience of physical activity and exercise.

### Reported Outcome Measures

A variety of outcome measures were reported across studies to explore the effects of PIMSs on physical activity and exercise-related behaviors. The outcome measures included assessments of physical activity levels, such as accelerometer-based metrics and adherence to specific heart rate zones, as well as psychological and perceptual outcomes such as mood (measured through tools such as the MDMQ and FS) and intrinsic motivation (measured via the Intrinsic Motivation Inventory, IMI). The RPE was frequently captured using the Borg Category-Ratio 10 Scale [51,52]. Table 3 presents this information. Further information on these outcome measurements can be found in Section S2 in Multimedia Appendix 1.

Studies used diverse technologies and protocols to assess PIMSs’ effects on physical activity and exercise behaviors. Reported technologies included accelerometers, heart rate monitors, and systems such as JYMMiN, which integrate real-time musical feedback with gym equipment. Analytical methods, such as ANOVA and multivariate analysis of variance, were used to evaluate outcomes, with specific systems adapting music based on cadence, heart rate, and intensity. Table 4 presents a detailed overview of these technologies and protocols.

**Table 3.** Outcome measures as reported in the studies.

Outcome measure and measurement method		Study
Physical activity level		
	Mean weekly minutes of physical activity measured using a triaxial accelerometer.	[29]
	Duration of exercise until exhaustion, timed with a stopwatch.	[22]
	Compliance with exercise regime by monitoring adherence to target heart rate zones during cycling.	[12]
Affective valence		
	Feeling Scale based on Russell circumplex model of affect.	[32]
	Multidimensional Mood Questionnaire, evaluating “good versus bad” mood dimensions during acute physical exercise.	[22,35]
	Interest or enjoyment subscale of the Intrinsic Motivation Inventory for intrinsic motivation.	[12,66,67]
Ratings of perceived exertion	Borg Category-Ratio 10 Scale, with ratings of perceived exertion collected at specific time intervals during exercise.	[12,22,32]
Physical exertion		
	Heart rate measured using a Polar Verity Sense (Polar Electro) device based on photoplethysmography.	[32]
	Pace measured via swings per minute using smartphone accelerometer data.	[36]
	Heart rate measured using a Polar T61 (Polar Electro) heart rate belt.	[12]

**Table 4.** Technologies and analysis protocols of PIMSs^a^.

Type of PIMSs	PIMS description	Data analysis protocol	Reference
Accelerometer	Music synchronization with step cadence	N/A^b^	[36]
Heart rate monitor	Music tempo adjustments based on physiological data	ANOVA and MANOVA^c^	[32]
JYMMiN	Music feedback system	ANOVA, MANOVA, and Wilcoxon Signed-Ranks Test	[22,35]
Magnet and heart rate sensors	Magnet sensors detect the ratings of perceived exertion, paired with heart rate, to optimize cycling rhythms	N/A	[37]
moBeat	Music feedback system	ANOVA	[12]
Musical sonification systems	Converts movement data into sound to enhance engagement and differentiate activity patterns	One proportion z-test	[39]
Musical sonification (custom pedals)	Pedals with load sensors and a microcontroller adjust musical feedback	ANOVA, Friedman test, and pairwise comparisons	[38]
Personal activity monitor	N/A	Generalized linear modeling	[29]
Three-axis accelerometer (smartphone)	Adjusts music tempo to synchronize with the user’s pace using swings per minute	N/A	[36]
Triaxial accelerometer	Uses accelerometer and heart rate to adjust music tempo for maintaining the target heart rate during cardio training	N/A	[33]

a PIMS: Personalized Interactive Music System.

b N/A: not applicable.

c MANOVA: multivariate analysis of variance.

### Risk of Bias in Studies

**Figure 2.**
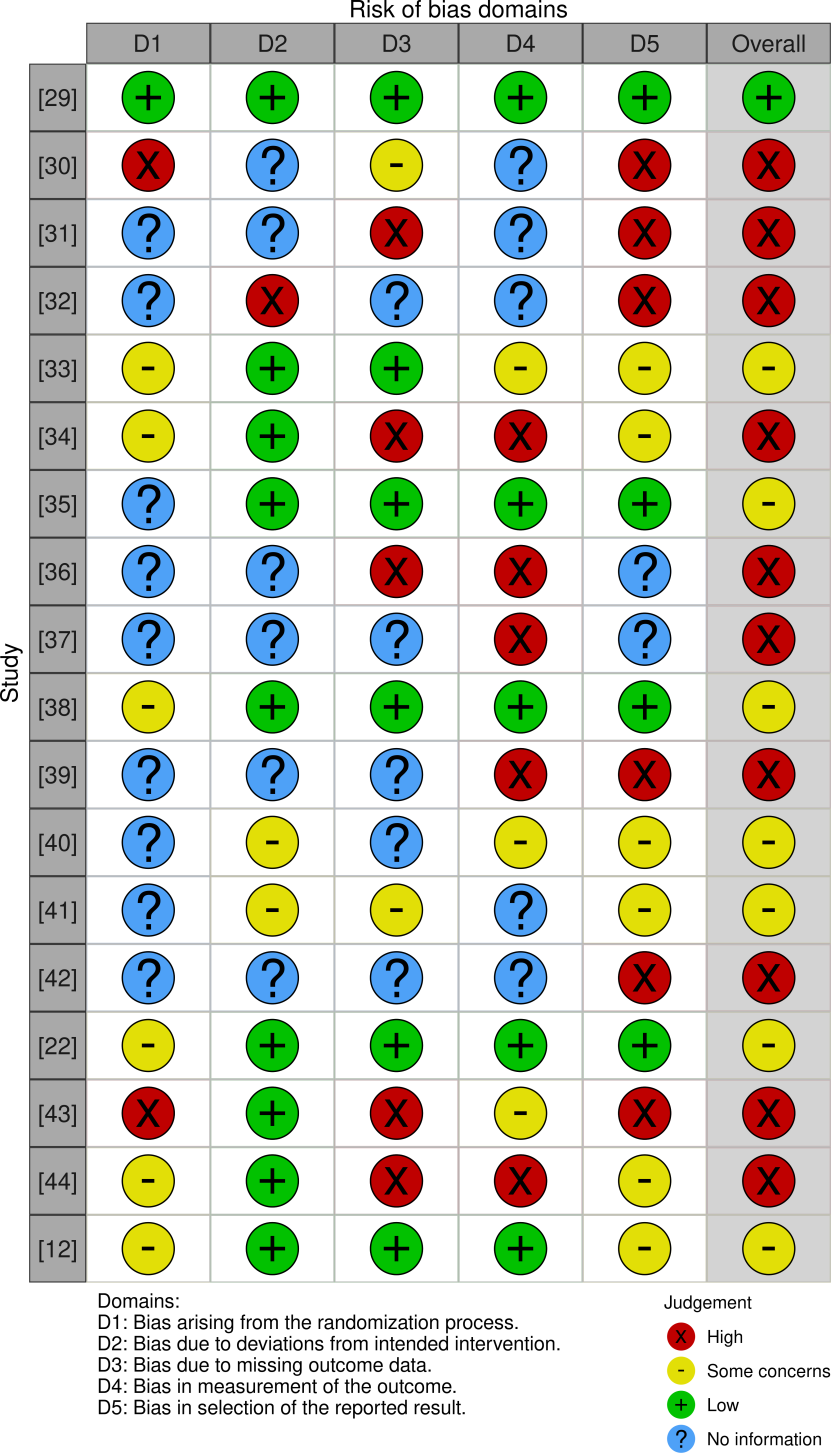
Evaluation of risk of bias in the included studies, categorized across 5 domains from D1 to D5 (cf [68]). An overall bias risk assessment for each study is also provided, conservatively summarizing the findings across all 5 domains [12,22,29-44]. D: domain.

Following the assessment of the study quality using the JBI critical appraisal checklist tools, the nine criteria were adapted to the 5 risk-of-bias domains found in the R package for risk-of-bias assessments (robvis) in the study by McGuinness and Higgins [68]. This assessment tool tests the risk of bias resulting from the randomization process (domain [D1]), deviations from intended intervention (D2), missing outcome data (D3), measurement of the outcome (D4), and selection of the reported result (D5). Each domain is assessed with a judgment scale indicating a high risk of bias (red cross), some concerns (yellow circle), low risk of bias (green plus), and no information (blue question mark; cf Figure 2).

We included all 18 studies in the review regardless of their overall risk of bias rating (see Figure 2, column “overall”). The overall risk of bias rating for each study was assigned conservatively, reflecting the highest risk level present across any of the 5 domains (D1-D5). For example, if 1 domain was judged to have a high risk of bias, the overall rating for that study was classified as high risk. Of the 18 studies, 1 randomized experimental design study [29] was rated for low risk of bias. Seven studies received a moderate (some concerns) rating of risk of bias, and 10 were rated for a high risk of bias.

### PIMSs Used in Experimental Studies

PIMSs were explored in experimental studies for their influence on physical, psychophysical, and affective exercise–related outcomes. Several studies focused on synchronization and auditory-motor coupling. Moens et al [41] examined beat synchronization using the D-Jogger adaptive music player. They found that initiating music in phase synchrony significantly enhanced consistent sensorimotor patterns, while strategies relying on tempo adjustments alone were less effective. Maes et al [38] provided detailed analyses of synchronization strength using SoundBike (Ghent University), where musical sonification significantly increased pedal cadence synchronization with external music. Similarly, Jun et al [36] found significant increases in step frequency (swings per minute) when music tempo aligned with user pace, enhancing consistency and efficiency of the activity.

Rehfeld et al [22] and Fritz et al [35] reported on the JYMMiN system’s role in improving mood and exercise duration. Notably, Fritz et al [35] observed mood enhancements in younger adults, while [22] noted prolonged exercise durations in older adult participants despite no significant mood changes. This may be potentially due to age-related differences in energy pacing. Rosseland [44] explored a tempo-responsive system for Alzheimer patients, observing improved synchronization and engagement. Additionally, van der Vlist et al [12] reported the moBeat system maintained exercise compliance while enhancing intrinsic motivation and attentional dissociation from discomfort. Sample sizes varied (N*=*10 to N=150), with participants aged 18‐79 years across diverse demographics. Detailed descriptions of PIMSs used in these studies can be found in Table S1 in Multimedia Appendix 3.

### PIMSs Used in Proof of Concept and User Testing Studies

Proof-of-concept and user-testing studies used PIMSs to adapt music or audio feedback based on real-time physical activity and exercise-related data (eg, heart rate, oxygen saturation, and galvanic skin response), with a focus on music recommendation systems and synchronization features. Álvarez et al [30] tested DJ-Running (University of Zaragoza), which integrates environmental (GPS) and galvanic skin response data to provide personalized music recommendations using algorithms such as artificial neural networks. Ospina-Bohórquez et al [42] developed a context-aware recommender system using smartphone sensors to adjust music based on exercise intensity, providing evidence for preliminary efficacy in low-concentration activities (eg, low-to-moderate intensity activities that require minimal concentration, such as walking).

Two synchronization-based systems were included: [33] music-assisted run trainer, which adjusts music tempo to heart rate or step frequency; and [31] music feedback exercise system, which synchronizes music with movement intensity through advanced audio processing. For example, as exercise intensity increases, additional layers of musical elements such as rhythm guitar, bass, or drums are progressively added to the audio track. Mendoza et al [39] introduced musical sonification, converting movement data into music for users to identify different physical activity patterns. Maculewicz and Serafin [37] examined ecological soundscapes to influence cycling behavior. Soundscapes were, for example, dynamically altered based on users’ cycling speed and heart rate.

Moens et al [40] reported optimal movement entrainment at ~120 BPM using D-Jogger but noted disruptions during song transitions. The reinforcement learning–based system by Fang et al [34] found improved user satisfaction and fewer track rejections, while Rosseland [44] found tempo-responsive music systems beneficial for older adults with Alzheimer disease. Details on these systems can be found in Table S2 in Multimedia Appendix 3.

### Meta-Analyses

A single overall meta-analysis of the studies was not achievable due to heterogeneity across datasets and outcomes [69]. Instead, the outcomes were reported separately based on their focus. The reported outcomes distinguished between (1) physical activity levels, (2) physical exertion, (3) RPE, and (4) affective valence.

### Results for Physical Activity Level

**Figure 3.**
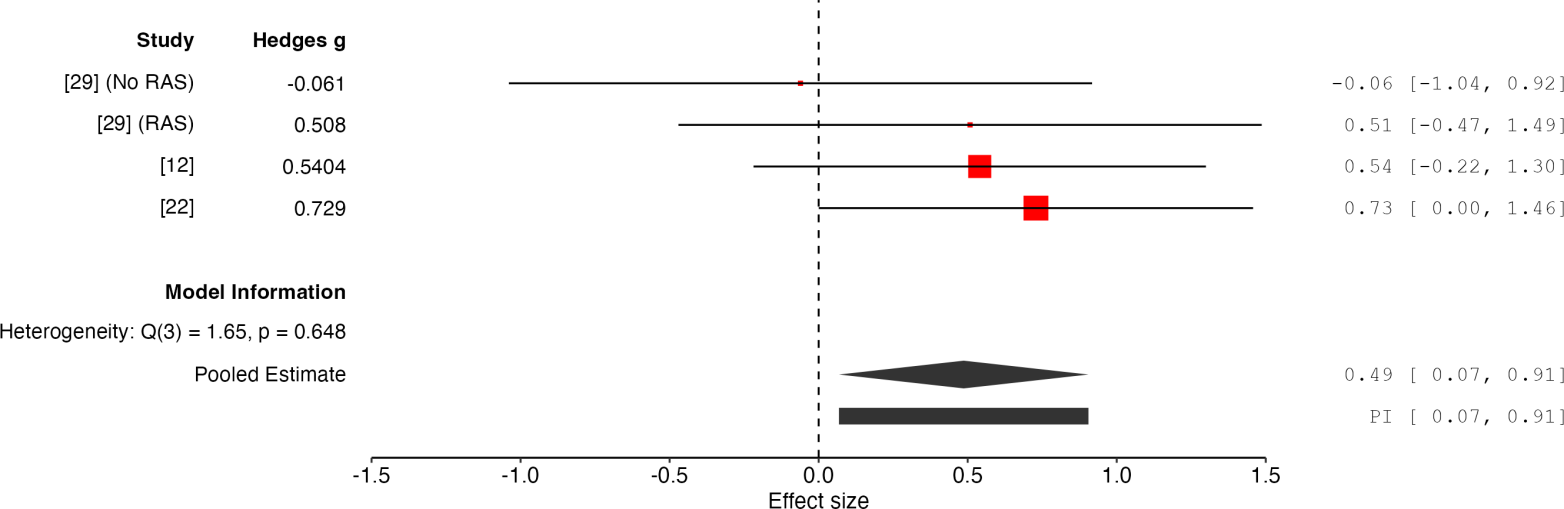
Forest plot of effect sizes for physical activity level outcomes associated with PIMSs [12,22,29]. PIMS: Personalized Interactive Music System; RAS: rhythmic auditory stimulation.

The overall effect size is 0.49 with a 95% CI of 0.07 to 0.91, and a *P* value of .02 (*k*=4, n*=*76). This indicates that the results are statistically significant, supporting the effectiveness of PIMSs in improving outcomes relating to physical activity level (Figure 3). The random-effects model indicates low heterogeneity (*Q*=1.65, *P*=.65, *I*²=0%, *τ*^2^=0.00) between the studies, suggesting it to be negligible. The calculated 95% prediction interval for the true effect size is 0.07 to 0.91, indicating that while the average effect is positive, the range of potential true effects across future studies could include larger positive outcomes.

### Results for Physical Exertion

**Figure 4.**
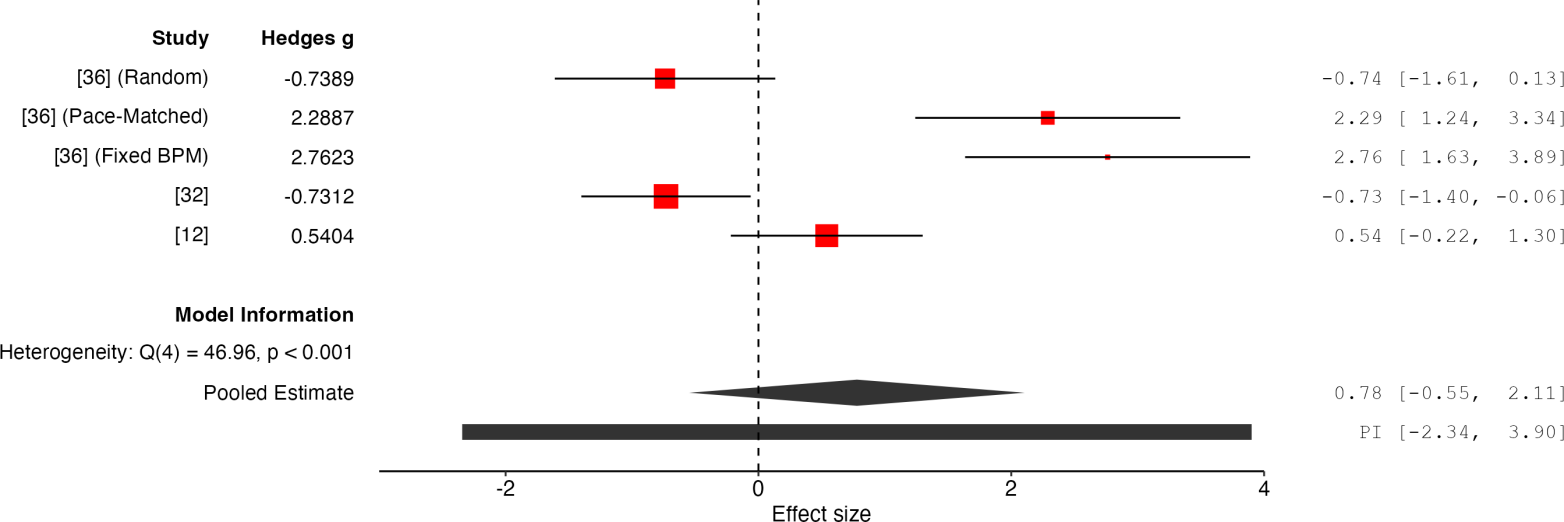
Forest plot of effect sizes for physical exertion outcomes associated with PIMSs [12,32,36]. BPM: beats per minute; PIMS: Personalized Interactive Music System.

The overall effect size is 0.78 with a 95% CI of −0.55 to 2.11, and a *P* value of .25 (*k*=5, n=142), indicating that the results are not statistically significant and do not support the effectiveness of PIMSs in improving physical exertion outcomes (Figure 4). The random-effects model indicates high heterogeneity (*Q*=46.96, *P*≤.001, *I*²=91%, *τ*^2^=2.08) between the studies. The calculated 95% prediction interval for the true effect size is −2.34 to 3.90, indicating the potential for considerable variation in the effects of PIMSs on physical exertion across future studies.

### Results for RPE

**Figure 5.**
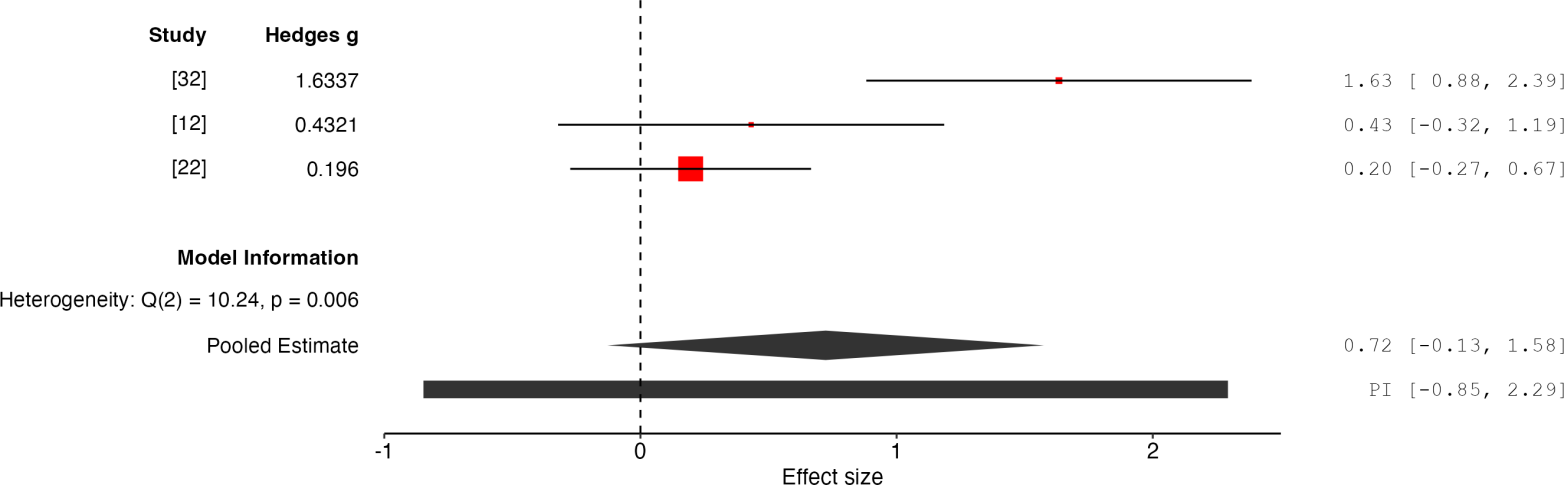
Forest plot of effect sizes for RPE outcomes associated with PIMSs [12,22,32]. PIMS: Personalized Interactive Music System; RPE: ratings of perceived exertion.

The overall effect size is 0.72 with a 95% CI of −0.13 to 1.58, and a *P* value of .10 (*k*=3, n=77), indicating that the results are not statistically significant and do not conclusively support the effectiveness of PIMSs in improving RPE outcomes (Figure 5). The random-effects model indicates substantial heterogeneity (*Q*=10.24, *P*=.01, *I²*=80%, *τ^2^*=0.45) between the studies. The calculated 95% prediction interval for the true effect size is −0.85 to 2.29, reflecting the significant variability in potential outcomes across future studies.

### Results for Affective Valence

**Figure 6.**
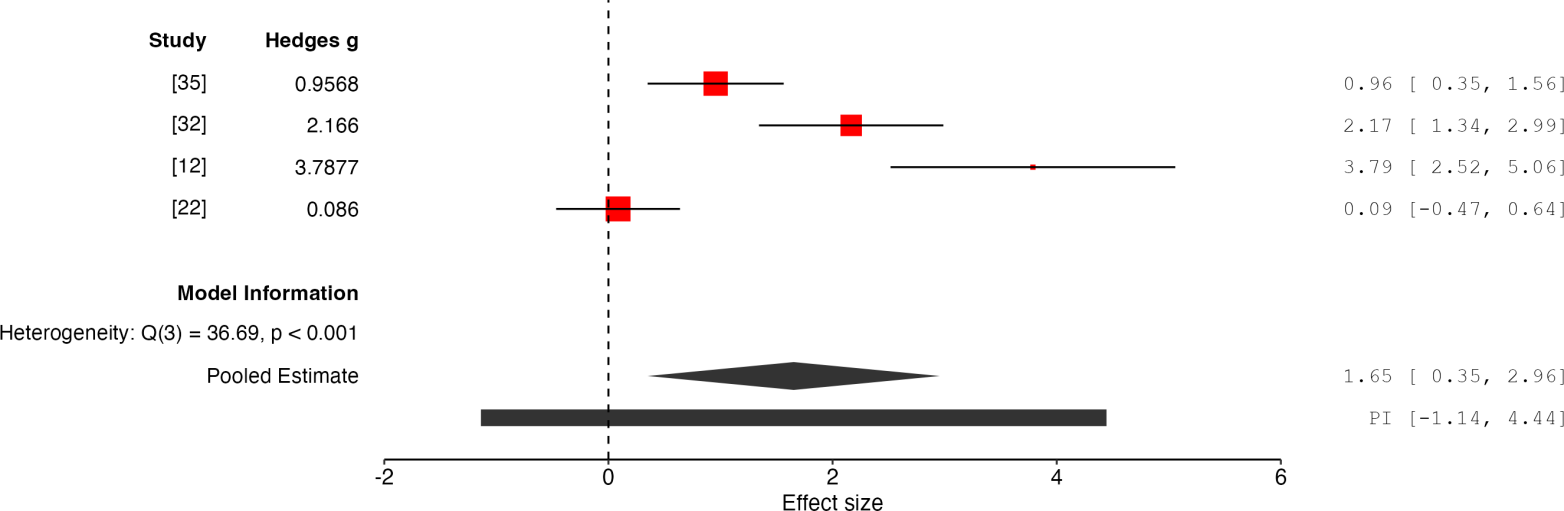
Forest plot of effect sizes for affective valence outcomes associated with PIMSs [12,22,32,35]. PIMS: Personalized Interactive Music System.

The overall effect size is 1.65 with a 95% CI of 0.35 to 2.96, and a *P* value of .01 (*k*=4, n=122), indicating that the results are statistically significant and thus consistent with the effectiveness of PIMSs in improving affective valence outcomes (Figure 6). The random-effects model indicates substantial heterogeneity (*Q*=36.69, *P*<.001, *I*²=92%, *τ*^2^=1.59) between the studies. The calculated 95% prediction interval for the true effect size is −1.14 to 4.44, highlighting significant variability in potential outcomes across future studies.

### Meta-Regression Analysis

**Figure 7.**
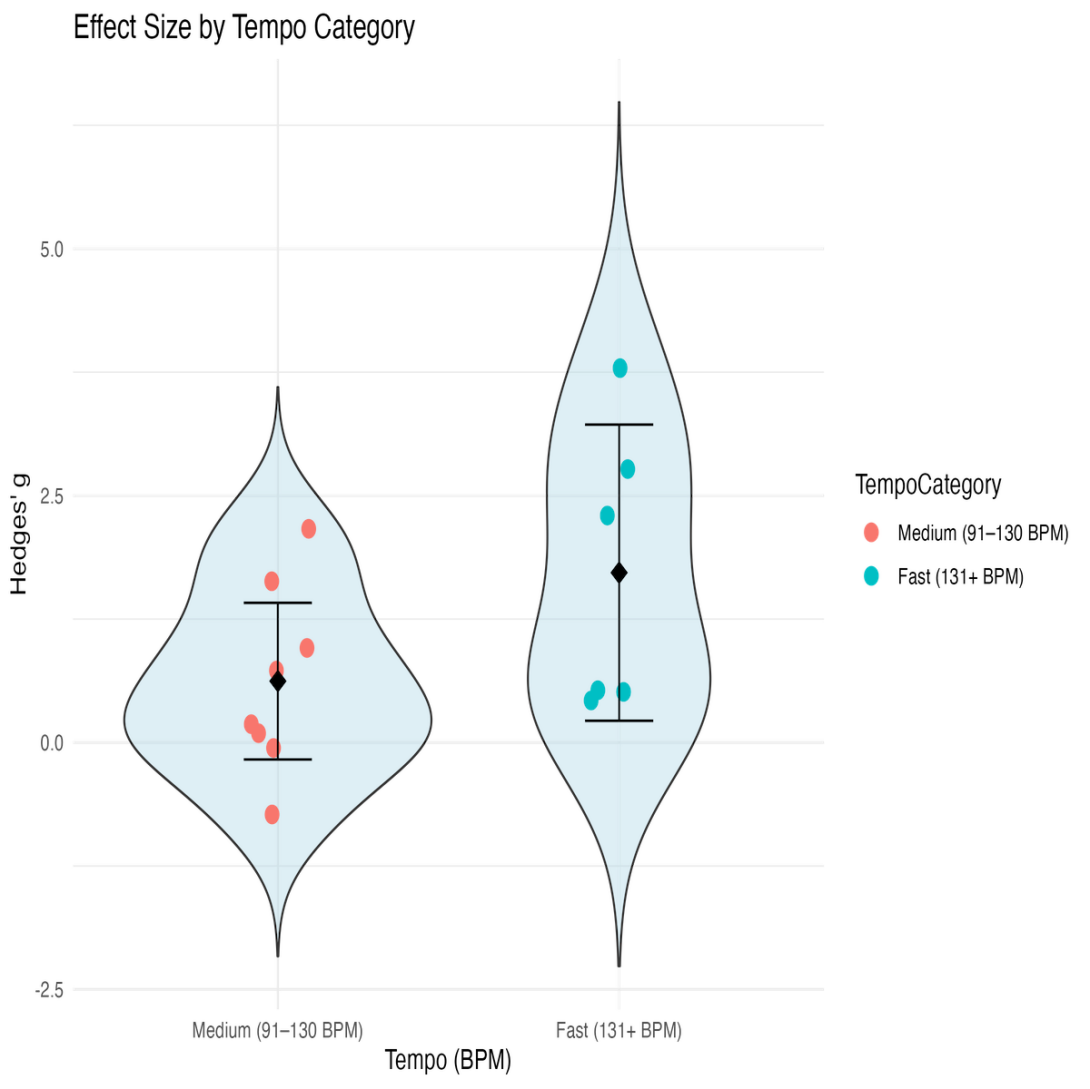
Distribution of study-level effect sizes across music tempo categories. Violin plots illustrate the density and spread of Hedges *g* values for medium (91‐130 BPM) and fast (131+ BPM) tempo groups. Dots represent individual study estimates; diamonds and error bars indicate group means and 95% CIs, respectively. BPM: beats per minute.

Heterogeneity was identified in the meta-analyses, prompting the use of meta-regression analysis to explore potential moderators of effect sizes. Music tempi showed a statistically significant positive association with effect sizes (*β*=.62, SE=0.29, *P*=.031), suggesting that faster tempi may have a significant effect across the outcomes of interest. None of the other predictors, including participant age, exercise intensity, or sample size, demonstrated a significant effect on effect sizes (Table 5 and Figure 7). The overall meta-regression model was not statistically significant, *Q_M_*(4)=7.03, *P*=.135, and a substantial portion of heterogeneity remained unexplained, *Q_E_*(11)=76.78, *P*<.001, *I*^2^=85.67%, *τ*^2^=0.92. This indicates that other, unexplored factors likely contribute to the variability in outcomes. Given the inclusion of all outcomes of interest in this analysis, the potential for residual variability and unaccounted-for heterogeneity is high.

**Table 5.** A summary of the meta-regression analysis.

Predictor	Estimate	SE	z	*P* value	95% CI	
Intercept	1.194	2.965	0.402	.69	−4.619 to 7.006	
Age	−0.035	0.040	−0.878	.38	−0.114 to 0.043	
Music tempo	0.617	0.286	2.155	.031	0.056 to 1.178	
Exercise intensity	0.212	1.262	0.168	.87	−2.262 to 2.686	
Sample size	−0.025	0.062	−0.402	.69	−0.147 to 0.097	

### Publication Bias Analysis (Egger Test)

**Figure 8.**
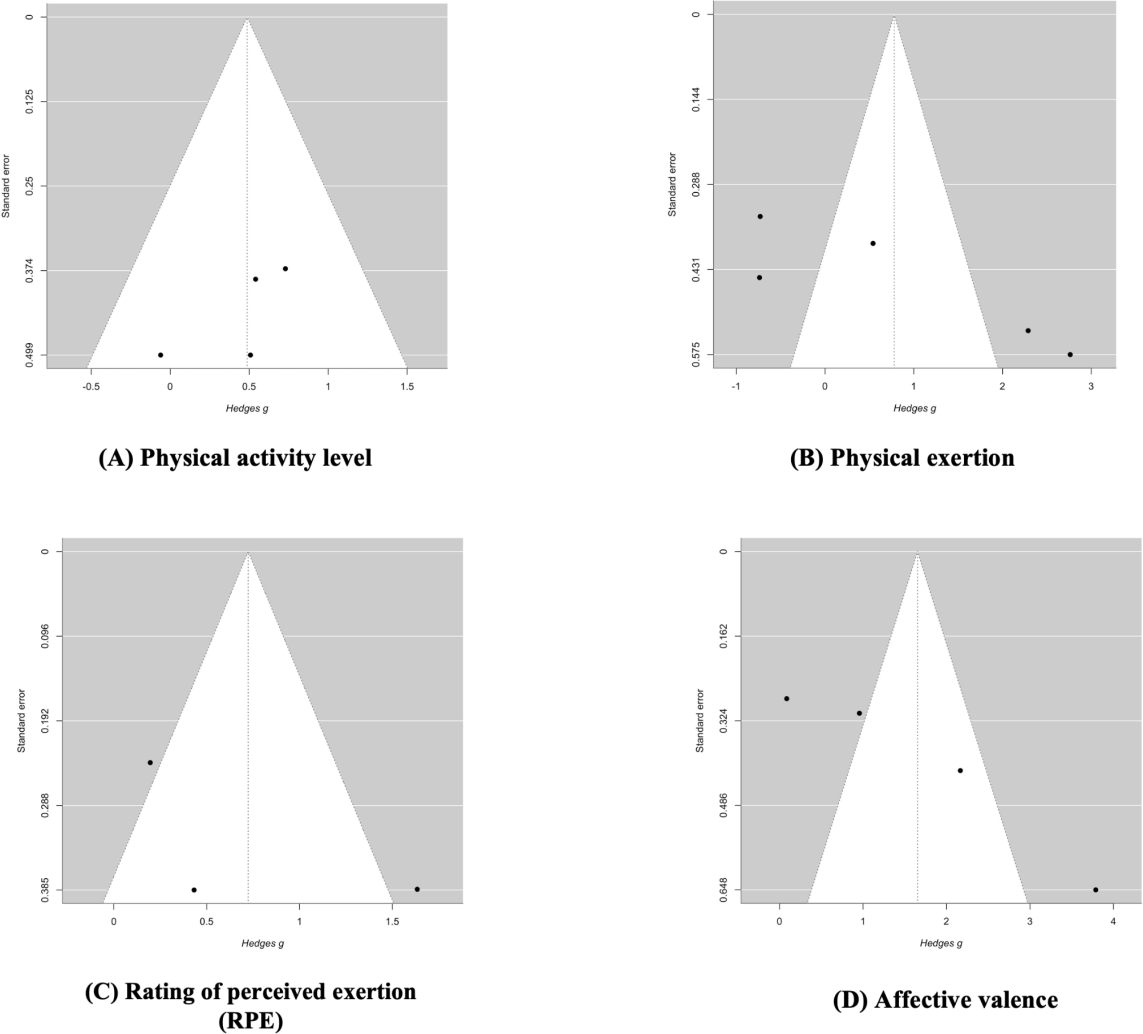
Funnel plots for (A) physical activity level, (B) physical exertion, (C) RPE, and (D) affective valence. RPE: ratings of perceived exertion.

Egger test [70] indicated nonsignificant asymmetry for physical activity level (*z*=−0.968, *P*=.333), significant asymmetry for physical exertion (*z*=2.927, *P*=.003), nonsignificant asymmetry for RPE (*z*=0.832, *P*=.405), and significant asymmetry for affective valence (*z*=4.961, *P*<.001; Figure 8). Due to potential publication bias, the summary effect sizes for physical exertion and affective valence outcomes may thus be slightly inflated.

## Discussion

### Principal Findings

This review aimed to systematically evaluate the effectiveness of PIMSs across physical activity levels, physiological outcomes (eg, heart rate and step frequency), psychophysical outcomes (eg, the RPE), and affective valence in relation to physical activity and exercise behaviors. A central focus was an exploratory meta-analysis of PIMSs across these outcome domains.

The exploratory meta-analysis revealed that PIMSs demonstrate favorable effects on physical activity levels and affective valence, with effect size estimates surpassing those of general music listening [6]. However, the certainty of evidence is limited by methodological inconsistencies, a moderate to high risk of bias, and the limited number of published studies eligible for meta-analyses. Importantly, no significant effects were observed for RPE or measured physical exertion. This reflects variability in the psychophysical outcomes associated with interventions using PIMSs.

When examining the findings of individual studies separately, they offer preliminary evidence that PIMSs may improve physical, psychophysical, and affective outcomes related to physical activity and exercise. For example [22], observed longer exercise durations during sessions using JYMMiN compared to routines with passive music listening, without significant increases in perceived exertion. Similarly, Alter et al [29] reported increased weekly physical activity volumes among cardiovascular disease patients using personalized rhythmic auditory stimulation–enhanced playlists. Additionally, Ren et al [43] provided qualitative evidence suggesting that PIMSs may prompt physical activity, such as reducing sitting time in office settings.

However, interpreting these findings is challenging due to methodological limitations and variability in population characteristics. Some studies focus on clinical populations, such as cardiovascular disease patients [29], while others target healthy younger adults [33] or older adult participants [22]. Several studies lack demographic details entirely, further complicating the assessment of population-specific efficacy. Sample sizes also vary widely, from single participants [31] to larger groups (N=36) [42].

### Heterogeneity in PIMSs’ Outcomes: Methodological Influences

The wide prediction intervals observed across outcome domains reflect the substantial heterogeneity in PIMSs’ effects. For example, prediction intervals for physical activity levels and affective valence highlight significant variability in potential effect sizes. This suggests that while PIMSs may provide positive average effects, individual study outcomes could range from substantial benefits to negligible or even negative impacts. Similarly, the prediction intervals for RPE and physical exertion emphasize uncertainty surrounding these psychophysical outcomes, pointing to inconsistencies in measurement and intervention design.

Specifically, variations in study methodologies and control group conditions contribute significantly to this heterogeneity. Some studies used passive music or other auditory stimuli as controls, while others used no-music conditions. This negatively affects comparability. Well-powered randomized designs, such as that by Alter et al [29] produced robust findings, whereas smaller studies, such as that by Rehfeld et al [22] yielded nonsignificant results, pointing to the influence of study design and statistical power. Additionally, short intervention durations and small sample sizes [37,39] constrain the generalizability of findings. The absence of standardized metrics and protocols across studies further hinders the ability to synthesize outcomes and develop systematic guidelines for PIMS interventions. To alleviate this, future research should adopt standardized protocols and outcome measures. This could be achieved via a music selection and delivery protocol, ensuring uniformity through a predefined library of music tracks categorized by tempo and intensity, delivered via standardized systems (eg, wireless headphones at consistent volumes). Validated tools such as the Borg RPE and the FS for measuring affective valence, administered at fixed intervals, may enhance comparability.

### Feasibility of PIMSs on Physical Activity Levels and Affective Outcomes

Despite methodological inconsistencies, our findings suggest that PIMSs may have a positive influence on physical activity levels. Studies in this cluster were rated as having low [29] to moderate [12,22] risk of bias, with both the studies by Alter et al [29] and Rehfeld et al [22] focusing on older adult populations. Positive effects include increased exercise duration (~66 seconds) [22] and overall weekly physical activity (~105.4 additional minutes per week on average) [29]. However, van der Vlist et al [12] found no significant impact of PIMSs on physical activity levels. The low heterogeneity in this cluster indicates consistent findings despite variations in study design and participant populations. This is promising and calls for further investigation.

Our results align with that of Clark et al [6], who noted that music listening, when combined with physical activity, enhances exercise outcomes in older adults. Both Alter et al [29] and van der Vlist et al [12] used synchronization strategies—rhythmic auditory stimulation and auditory-motor coupling, respectively—consistent with frameworks by Bood et al [8] and Clark et al [71] that link synchronized music to improved physical activity and exercise performance. However, the exploratory nature of the meta-analysis and the small number of studies limit the potential generalizability of these findings. Further research with diverse populations and robust methodologies is required to confirm whether PIMSs are effective adjuncts for increasing physical activity levels.

For affective valence, the large effect size estimate suggests PIMSs contribute to elevated affective experiences during physical activity and exercise [12,22,32,35]. However, this finding is strongly influenced by van der Vlist et al [12], whose notably high effect size estimate substantially raised the overall meta-analytic effect size estimate. In contrast, smaller effects observed in other studies [22,35] reduced the precision and generalizability of the overall meta-analytic finding. The differences in these outcomes likely reflect variations in music selection methods: researcher-selected music in the study by van der Vlist et al [12] prompted synchronization and enjoyment (“fun and enjoyment” ratings via IMI), while self-selected music [32] and device-generated feedback [22,35] influenced affective outcomes in distinct ways. In the study by van der Vlist [12], researcher-selected music facilitated synchronization, while Chen et al [32] used self-selected music based on participants’ individual preferences. Rehfeld et al [22] and Fritz et al [35] used device-generated musical feedback, where participants’ movements influenced the music. These differences suggest that PIMSs may enhance affective valence outcomes during physical activity and exercise through both self-selected and researcher-selected music, with evidence of positive effects for music tailored to individual preferences (aligning with prior research by Terry et al [7] and Khalfa et al [11]) as well as for standardized, researcher-selected stimuli.

Curiously, van der Vlist et al [12] reported no significant benefits for RPE, despite using auditory-motor coupling strategies. This discrepancy may find alignment with the Dual-Mode Theory, as even though music can enhance automatic synchronization and facilitate improved physical performance, it does not always mitigate RPE if reflective processes (eg, cognitive appraisal of effort) are less engaged [13]. The substantial heterogeneity within the affective valence cluster, driven by variability in musical strategies, participant demographics, and inconsistent measurement tools (eg, MDMQ, IMI, and FS), further supports ART’s assertion that individual and contextual factors critically shape affective outcomes during exercise.

All studies in the affective valence cluster were deemed to have a moderate risk of bias. Furthermore, the reliance on measurement scales without strong theoretical grounding, as noted in the study by van der Vlist et al [12], suggests the need for alignment with validated frameworks such as ART. For instance, the FS used by Chen et al [32] directly measures the pleasure-displeasure dimensions central to ART, aligning with validated frameworks in physical activity and exercise contexts [72]. The FS provides a theoretically robust and context-specific assessment of affective responses, capturing the transient emotional states during exercise that ART posits are critical for shaping future behavioral intentions. These findings tentatively indicate that these PIMSs leverage momentary affective responses to improve exercise experiences [6,7,17]. In sum, findings across the physical activity and affective valence meta-analytic clusters suggest PIMSs may support affect augmentation during physical activity, highlighting their potential to enhance both physical activity levels and affective outcomes [5,17].

### PIMSs’ Tempo Adjustments and Synchronization in Physical Activity and Exercise Outcomes

The identification of faster music tempi as a statistically significant moderator in the meta-regression aligns with evidence supporting the role of synchronization strength and auditory-motor coupling in enhancing exercise outcomes [8,60]. For instance, faster tempi provide consistent rhythmic cues that facilitate the alignment of motor actions with auditory stimuli. This can optimize auditory-motor coupling [8-10], which, in turn, enables predictive synchronization to reduce RPE [7]. For example, Chen et al [32] reported that real-time tempo adjustments based on heart rate significantly reduced RPE and improved affective responses. This indicates that synchronized music facilitated participants’ dissociation from internal sensory signals and promoted enjoyment during exercise [7].

### Limitations and Future Directions

This review presents the first systematic exploration of PIMSs exclusively within physical, psychophysical, and affective domains of physical activity and exercise. While it provides valuable insights, several limitations must be acknowledged. A significant proportion of the included studies (14 of 18) primarily assessed the feasibility of PIMSs, with few investigating direct outcomes related to physical activity or exercise. Many experimental studies were limited by short durations, small sample sizes, and insufficiently rigorous methodologies. Similarly, proof-of-concept and user-testing studies largely focused on system feasibility rather than assessing objective psychophysiological outcomes. Consequently, the high risk of bias in 10 studies underscores the overall low quality of evidence. Additionally, the small number of eligible studies precluded sensitivity analyses, which further emphasizes the preliminary nature of this review’s findings.

Few studies identified physical activity as a primary outcome, often relegating it to secondary importance. Objective assessments of physical activity—such as measures of frequency, intensity, and duration—were notably absent, making it difficult to draw robust conclusions or compare results across studies. Standardizing methods for quantifying physical activity would enhance future research by enabling more meaningful cross-study comparisons.

Furthermore, the methodology used in this study was limited by substantial heterogeneity across studies. This prevents a unified meta-analysis and necessitates the reporting of separate outcomes. Variability in study designs, participant demographics, and measurement tools contributed to unexplained heterogeneity, while the small number of studies precluded sensitivity analyses. These factors, combined with the exploratory nature of the meta-analysis, point to the need for standardized methodologies and rigorous reporting in future research. Additional limitations include the possibility of publication and retrieval bias, as only English-language studies from selected databases were included. Furthermore, although screening and data extraction were independently conducted by 2 reviewers, the use of automated tools and subjective judgment may have introduced bias.

To address these limitations, future research should prioritize larger, randomized controlled trials with diverse populations and longer intervention periods. Longitudinal studies are particularly needed to evaluate the sustained impact of PIMSs on physical activity and exercise. Additionally, investigating the mechanisms underlying individual variability in PIMSs’ responses could optimize these systems for different populations and exercise contexts. This highlights the need for more rigorous research to validate these effects and refine PIMSs’ interventions, particularly through the development of dynamic systems that can adapt tempo in real time to suit diverse user needs and exercise contexts [41,71].

Emerging trends in PIMSs, such as music recommender systems examined by Álvarez et al [30], Fang et al [34], and Ospina-Bohórquez et al [42], highlight the potential for integration with streaming services such as Spotify (Spotify AB). These systems demonstrated promising user feedback [34] and feasibility, suggesting they could serve as a foundation for future hypothesis-driven studies. Incorporating feedback from wearable and smartphone devices offers another avenue for development, allowing PIMSs to adapt based on physical activity and exercise metrics as well as music preferences. Finally, many PIMSs are relatively low-cost interventions (eg, the devices in the study by Alter et al [29] cost approximately US $75 per patient) and could have significant cost-effectiveness implications as part of broader health policy strategies to enhance physical activity and exercise participation at the population level [5].

### Conclusions

This systematic review provides exploratory evidence that PIMSs may positively impact physical activity levels and affective valence in physical activity and exercise contexts. The meta-analysis revealed moderate effect sizes for physical activity levels and significant but heterogeneously distributed effects for affective valence. However, outcomes for RPE and physical exertion were inconclusive due to high heterogeneity and limited study quality.

The findings are constrained by methodological limitations, including high risk of bias, small sample sizes, short study durations, and inconsistent measures across studies. Furthermore, the lack of theoretical frameworks for informing PIMSs’ designs and the absence of standardization in quantifying physical activity outcomes limit the generalizability of these findings. PIMSs remain considerably underexplored, and further research is essential.

Overall, PIMSs provide promising potential for enhancing physical activity levels and elevated affective valence, offering engaging physical activity and exercise opportunities for the public at large. With advancements in adaptive systems capable of real-time tempo adjustments, PIMSs may emerge as effective adjuncts for physical activity and exercise, pending rigorous validation in diverse populations.

## Supplementary material

10.2196/70372 Checklist 1 PRISMA checklist. PRISMA: Preferred Reporting Items for Systematic Reviews and Meta-Analyses.

10.2196/70372 Multimedia Appendix 1 Descriptions and outcomes of the PIMSs used across studies in sections. PIMS: Personalized Interactive Music System.

10.2196/70372 Multimedia Appendix 2 Data and syntax for meta-analysis.

10.2196/70372 Multimedia Appendix 3 Descriptions and outcomes of the PIMSs used across studies in tables. PIMS: Personalized Interactive Music System.

## Abbreviations

ART: Affective-Reflective Theory
BPM: beats per minute
D: domain
FS: Feeling Scale
IMI: Intrinsic Motivation Inventory
JBI: Joanna Briggs Institute
MDMQ: Multidimensional Mood Questionnaire
MET: metabolic equivalent of task
PIMS: Personalized Interactive Music System
RPE: ratings of perceived exertion
