# Supplementary material for: Personalized Interactive Music Systems for Physical Activity and Exercise: Exploratory Systematic Review and Meta-Analysis
Source: JMIR Hum Factors. 2025 Sep 8;12:e70372. doi: 10.2196/70372 (PMC12422526; doi:10.2196/70372)
Supplement: Multimedia Appendix 1 — Descriptions and outcomes of the PIMSs used across studies in sections. PIMS: Personalized Interactive Music System. [file humanfactors-v12-e70372-s002.docx]

**Section S1.** Operationalisation of terms.

- **Physical activity level** encompasses the volume, intensity, and compliance with physical activity recommendations or exercise regimens. Volume represents the overall amount of physical activity, and can be measured using metrics such as mean total counts per day [29]. Intensity can be categorised as absolute intensity using metabolic equivalents (METs) or relative intensity expressed as a percentage of an individual’s maximum oxygen uptake reserve (%VO2R, [30]). Compliance, referring to adherence to recommendations or regimens, can be assessed by tracking changes in physical activity volume, monitoring device usage, or measuring adherence to target heart-rate zones during physical activity or acute bouts of physical exercise [29].
- **Affective valence** refers to the emotional response of pleasure or displeasure experienced during or after physical activity. It focuses specifically on the valence dimension of emotional states—how positive or negative the feeling is—without incorporating arousal (activation). Affective valence captures moment-to-moment changes in an individual’s subjective emotional state as they engage in physical activity. For instance, physical activity often elicits feelings of pleasure (positive affect), though the intensity and context of the activity, as well as individual differences, influence these responses.
- **Rating of perceived exertion (RPE)** is a subjective numerical value that individuals assign to their sense of how hard their body is working during physical activity [7,37]. It is formed from a multitude of sensory cues, integrating both physiological sensations and psychological perceptions [37]. Sensations from muscles, skin, and joints, and effects stemming from the cardio-pulmonary system all contribute to this overall perception. The Borg RPE scale is specifically designed to measure perceived exertion during steady-state aerobic exercise, such as cycling or running. The commonly used Borg CR-10 scale, is a category-ratio scale, designed to measure the perceived intensity of various sensations, experiences, and feelings. Its primary application is in assessing perceived exertion but can also gauge other subjective experiences.
- **Physical exertion** is the effort exerted by the body to perform physical activity, characterised by the physiological, biomechanical, and perceptual demands placed on the individual [35]. Heart rate is a fundamental physiological indicator of exertion, reflecting the cardiovascular system’s response to physical demands [35]. Stride length and pace are essential biomechanical parameters, particularly in activities such as running and walking [35].

**Section S2.** Description of the outcome measures used in the studies.

- **Physical Activity Level:** [54] measured physical activity level by mean weekly minutes of physical activity, which was captured using a tri-axial accelerometer worn by the participants. [22] measured physical activity level through the duration of exercise until exhaustion, timed with a stopwatch. [12] measured compliance with a prescribed exercise regime by monitoring participants' adherence to target heart rate zones during a cycling session.
- **Affective valence:** [56] measured affective valence using the Feeling Scale (FS). The FS is based on Russell's circumplex model of affect [14,33]. The FS assesses affective valence (how positive or negative someone feels). [22,34] used the Multidimensional Mood Questionnaire (MDMQ) [32] to study the effects of PIMS on mood during acute bouts of physical exercise. The MDMQ includes subscales for “good vs. bad mood," "calmness vs. agitation," and "alertness vs. tiredness." While the MDMQ’s three subscales generally correspond to the circumplex model of affect [14,33] only the "good vs. bad mood" subscale was used, as it specifically aligns with the pleasure-displeasure (affective valence) dimension of affect. [12] examined the effect of PIMS on intrinsic motivation using an “interest/enjoyment” subscale of the Intrinsic Motivation Inventory (IMI) [66] during acute bouts of physical exercise. While not a direct measurement of affect, this subscale reflects positive valence and enjoyment, aligning broadly with the pleasure (affective valence) dimension of affect [67].
- **RPE:** All three studies that assessed Rating of Perceived Exertion (RPE) used the Borg CR-10 scale to capture participants' subjective experience of exercise intensity. [22,56] both employed the scale, with [56] collecting RPE ratings at specific time intervals during the exercise, while [22] opted for more frequent measurements every 90 seconds and at the conclusion of the exercise. [12] also utilised the Borg CR-10 scale, though the precise timing of their measurements is not detailed.
- **Physical Exertion:**[56] measured heart rate as a physiological measure of exertion using a Polar Verity Sense upper-arm heart-rate measurement device based on photoplethysmography (PPG). [58] measured pace by calculating Swings Per Minute (SWPM) using data from the accelerometer on a smartphone. [12] measured heart rate via a Polar T61 heart rate belt.

**Section S3.** Full search strategies by database.

**Web of Science**

TS=(“Personali*ed Interactive Music System*” OR “Music Recommendation Algorithm” OR “Music Recommendation System*” OR “Streaming” OR “MP3” OR “Digital Music”) AND TS=(“Physical Activity” OR “Exercise” OR “Recovery” OR “Recuperation” OR “Sedentary Behav*” OR “Physical Inactivity”)

**SPORTDiscus**

TX (“Personali*ed Interactive Music System*” OR “Music Recommendation Algorithm” OR “Music Recommendation System*” OR “Streaming” OR “MP3” OR “Digital Music”) AND TX (“Physical Activity” OR “Exercise” OR “Recovery” OR “Recuperation” OR “Sedentary Behav*” OR “Physical Inactivity”)

**Medline**

("Personali*ed Interactive Music System*" OR "Music Recommendation Algorithm" OR "Music Recommendation System*" OR "Streaming" OR "MP3" OR "Digital Music") AND ("Physical Activity"[MeSH Terms] OR "Exercise"[MeSH Terms] OR "Recovery" OR "Recuperation" OR "Sedentary Behavior"[MeSH Terms] OR "Physical Inactivity")

**Embase**

('personalized interactive music system':ti,ab,kw OR 'music recommendation algorithm':ti,ab,kw OR 'music recommendation system':ti,ab,kw OR streaming:ti,ab,kw OR mp3:ti,ab,kw OR 'digital music':ti,ab,kw) AND ('physical activity'/exp OR exercise/exp OR recovery/exp OR 'sedentary behavior'/exp OR 'physical inactivity'/exp)

**ACM Digital Library**

("Personalized Interactive Music System" OR "Music Recommendation Algorithm" OR "Music Recommendation System" OR "Streaming" OR "MP3" OR "Digital Music") AND ("Physical Activity" OR "Exercise" OR "Recovery" OR "Recuperation" OR "Sedentary Behavior" OR "Physical Inactivity")

**SpringerLink**

("Personalized Interactive Music System" OR "Music Recommendation Algorithm" OR "Music Recommendation System" OR "Streaming" OR "MP3" OR "Digital Music") AND ("Physical Activity" OR "Exercise" OR "Recovery" OR "Recuperation" OR "Sedentary Behavior" OR "Physical Inactivity")

**Google Scholar**

allintitle: ("Personalized Interactive Music System" OR "Music Recommendation System") AND ("Physical Activity" OR "Exercise")

**IEEE Xplore**

("All Metadata":"Personalized Interactive Music System" OR "Music Recommendation Algorithm" OR "Streaming" OR "MP3" OR "Digital Music") AND ("Physical Activity" OR "Exercise" OR "Recovery" OR "Sedentary Behavior" OR "Physical Inactivity")

**Scopus**

TITLE-ABS-KEY("Personalized Interactive Music System" OR "Music Recommendation Algorithm" OR "Music Recommendation System" OR "Streaming" OR "MP3" OR "Digital Music") AND TITLE-ABS-KEY("Physical Activity" OR "Exercise" OR "Recovery" OR "Recuperation" OR "Sedentary Behavior" OR "Physical Inactivity")
