# Supplementary material for: Personalized Interactive Music Systems for Physical Activity and Exercise: Exploratory Systematic Review and Meta-Analysis
Source: JMIR Hum Factors. 2025 Sep 8;12:e70372. doi: 10.2196/70372 (PMC12422526; doi:10.2196/70372)
Supplement: Multimedia Appendix 2 — Data and syntax for meta-analysis. [file humanfactors-v12-e70372-s003.docx]

Data and syntax for meta-analysis available via the Open Science Framework (OSF): https://osf.io/jpy5k
