# Supplementary material for: Personalized Interactive Music Systems for Physical Activity and Exercise: Exploratory Systematic Review and Meta-Analysis
Source: JMIR Hum Factors. 2025 Sep 8;12:e70372. doi: 10.2196/70372 (PMC12422526; doi:10.2196/70372)
Supplement: Multimedia Appendix 3 — Descriptions and outcomes of the PIMSs used across studies in tables. PIMS: Personalized Interactive Music System. [file humanfactors-v12-e70372-s004.docx]

**Table S1.** Descriptions and outcomes of the PIMS used in the nine experimental studies.

| **Reference** | **PIMS Used** | **PIMS Description** | **Outcomes** |
| --- | --- | --- | --- |
| [29] | Music audio-playlists | Personalised music audio-playlists to improve adherence to physical activity among cardiovascular disease patients participating in a structured exercise program. | Control (No Music), 370.2 minutes, *P* < .001^a^  Music Playlists, 475.6 minutes, *P* < .001^a^  Music Playlists with RAS, 631.3 minutes |
| [33] | Music Assisted Run Trainer (MART) | A smartphone application, to assist jogging activity by adapting music tempo based on the user’s step frequency or heart rate. | Aligning music tempo with step frequency – targeting optimal heart rate for cardiovascular training. |
| [34] | Music Recommendation System | A music recommendation system to motivate individuals to exercise more effectively by incorporating user profiling and reinforcement learning. | Improved satisfaction with playlists, reduced number of rejections needed to finalise a playlist. |
| [35] | Jymmin® | Exercise with musical feedback | Significant main effect of the "jymmin" condition on mood enhancement vs. passive listening, with a reported F(1, 43) = 10.67, *P* < .05 |
| [38] | SoundBike | A stationary bicycle system designed to enhance cyclists' spontaneous synchronisation with external music through musical sonification | Sonification using a beep at the point of maximum pedal pressure significantly increased cyclists' synchronisation strength with external music compared to no sonification (*P* < .05) and improved pedal cadence stability (*P* < .05). The beep condition resulted in a medium to large effect size for synchronisation and stability compared to other conditions. |
| [41] | D-Jogger | An adaptive music player designed to align music with the user’s walking or running pace. | Alignment Strategy 1 – Minor increase in steps synchronised with beat.  Alignment Strategy 2 – Minor increase in steps synchronised with beat.  Alignment Strategy 3 –Tempo adjusts to match runner’s pace throughout. Increase in synchronised steps; high phase-lock stability.  Alignment Strategy 4 – Adjusts phase and tempo during song to match each beat to footfall. Increase in synchronised steps; highest phase-lock stability. |
| [22] | Jymmin® | Exercise with musical feedback | Participants exercised significantly longer with Jymmin® (Jymmin® workout 248.75 seconds vs. Conventional workout 182.73 seconds) |
| [43] | Flow Platform (Smart Cushion) | A flow platform (a smart cushion) which used interactive music to cue office workers to reduce their sedentary time. | Both interactive and continuous music were similarly effective in motivating posture changes and reducing sedentary behaviour. |
| [12] | MoBeat | Intensity-based coaching during exercise by giving real-time feedback on training pace and intensity through interactive music. | The moBeat system was found to have a significant positive effect on intrinsic motivation (*P* < .001) and attentional focus (*P* < .001) during exercise. The moBeat system did not significantly reduce perceived exertion (*P* = .266) compared to the reference system. |

^a^ = *P*-value compares music playlists vs. a control group with no music. The *p*-value for the RAS-enhanced group compared to the others is not specified here but indicated as significant in the study.

**Table S2.** Descriptions and outcomes of the PIMS used in the nine proof of concept and user testing studies.

| **Reference** | **PIMS Used** | **PIMS Description** | **Outcomes** |
| --- | --- | --- | --- |
| [30] | The DJ Running System | The system dynamically adapts music using sensor data from physical movements or exercise equipment. | N/A |
| [31] | Music Feedback Exercise (MFE) | The study integrates MFE into Soundjack, using sensors to modulate music playback based on exercise intensity. | Positive feedback on system responsiveness and music integration. |
| [32] | Music-Assisted Internet of Things (IoT) Exercise System | The system adapts music tempo in real-time to the runner's heart rate. | N/A |
| [36] | Runner’s Jukebox (RJ) | RJ uses a pace recognition algorithm enabling the music player to play songs matched with the user pace and adjust playback speed dynamically to follow the user’s pace changes. | Fixed BPM and pace matching gave better exercise effect (SWPM) vs. no music and vs. randomly selected music |
| [37] | A Stationary bike augmented in an audio reality environment. | The use of sensors to monitor the user's pace and heart rate while exercising on a stationary bike. Audio feedback and cues are manipulated based on the user's performance. | N/A |
| [40] | DJogger | A music interface to leverage body movement in order to select music and adapt its tempo to the user’s pace, focusing on entrainment – synchronisation between music and walking. | The majority of subjects synchronised to the beats, no matter what the users’ pace.  If the music tempo is close enough to the user’s pace, the user tends to synchronise his/her steps with the beats. |
| [39] | Sonification of accelerometry data (recordings of physical activity) | This system transforms daily physical activity data recorded by wearable devices into musical pieces. | The majority of participants correctly identified the musical sonification associated with physical activity. |
| [42] | Context-Aware Music Recommender System (CAMRS) | The system classified physical activities by analysing data from the smartphone's accelerometer, allowing it to predict the user's activity. It recommends music with a Beats Per Minute (BPM) value to match the intensity of the detected physical activity. | When using the system, rates of perceived effort decreased in the majority of cases, and mood improved in the majority of cases. |
| [44] | Interactive Music System for Alzheimer’s patients | This music system for Alzheimer's patients, focuses on how dynamically adapting musical beats and rhythms can stimulate and motivate physical activity. The system adapts the tempo of music to match the pace of repetitive bodily movements. | The interactive music system produces an entraining effect on participants. |
